# Intelligence

**Published:** 1823-09-01

**Authors:** 


### INTELLIGENCE.

*Annual Report of the General Committee of the Associated Apothecaries and Surgeon Apothecaries of England and Wales, received and adopted at the Annual General Meeting of the Association held by Public Advertisement, at the Crown and Anchor Tavern, Strand, July 2d,* 1823.

JOSEPH HAYES, Esq. *President.*

YOUR Committee, in compliance with annual custom, submit to the Association, the result of their proceedings since the last General Meeting.

The meetings of the Committee have been regularly held, although there has been but little business to transact, and that little of no particular interest or urgency.

The Committee have not, however, been indifferent to the objects for which the Association was formed, and in the first volume of Transactions, now nearly completed, although necessarily partaking of the imperfections incident to the commencement of a new undertaking, they trust will be found an earnest of the zeal which they entertain for the welfare of the Medical Profession, and of their sincere desire to render the exertions of its members subservient to the public good, and the best interests of humanity.

Viewing, as they do, with satisfaction, the success which has so far attended the exertions of this Association, in the improved state of medical education, particularly in that of the general practitioner, they still hope to witness further benefits, which cannot ultimately be otherwise than reciprocal between the profession and the public.

Well aware of the difficulties which attended the passing of the Apothecaries' Act, and of the opposing obstacles which prevented the fulfilment of the laudable wishes of the Association, with which it originated, and of the profession at large, in rendering it fully adequate to its proposed end, the Committee still cherish the hope of obtaining further improvements through the medium of the legislature, which, whilst they may afford the necessary protection of the various members of the profession, shall be founded on a more dignified and extensive basis—the public good.

They therefore abstain in the present instance, from any other than general allusion to the imperfections of the existing state of the Medical Profession, and of the legislative provisions relative to it, or the means required to affect the necessary meliorations. The work of improvement is slow and laborious, but when attempted with zeal, and carried on with perseverance, and with a purity of motive, which the end desired must show to be above suspicion, it cannot be otherwise than progressive.

They who now labour to place the practice of medicine upon a footing more consistent with its real dignity and usefulness, can scarcely expect personally to reap any benefit, unless the consciousness of having strenuously exerted themselves to effect the diminution of human suffering can be supposed to bear such an interpretation.

As an accurate knowledge of the present state of the medical profession can be the only ground-work of any rational attempt to effect improvement, the Committee beg leave to recommend to the Association that it be therefore an instruction to the future Committee—

1. To ascertain, as far as may be practicable, the actual condition of the Medical Profession, and the obstacles which have hitherto impeded its advancement.

2. To examine and to arrange the imperfections to which its present condition is liable, showing the particulars in which the public and members of the profession are injured or aggrieved.

3. To point out the remedies for the evils complained of.

4. To use such means, as may to them seem proper, to effect (whether by application to the legislature, or by other legal means) the meliorations to be desired.

5. To continue to collect and record the experience of the profession on subjects relating to Medical Science, and to publish the same in occasional or annual volumes, calculated to prove the just claims of the different classes of the practitioners of the healing art to that confidence of the public which they have so long enjoyed.

6. To give a brief account of the discoveries and improvements which have been made in medicine, surgery, and the accessory sciences, within the preceding year.

7. To offer such encouragements of honorary rewards, as may to them (the Committee) seem proper, for the best practical Essays on select subjects relating to Medicine and Surgery, as may call forth the energies, particularly of the younger members of the profession, and elicit improvements in the healing art.

8. That the profits arising from the publication of the Transactions of this Association, be devoted to the above-named laudable purpose.

Published by order of the Meeting,

1, Keppel Street. JOHN POWELL, *Secretary.*

---

An elderly married medical man, who is living in one of the best streets at the West End of the Town, and who is occupying a very large house, within five minutes walk of the Schools of Medicine and of Anatomy, receives into his house a limited number of medical students to board and lodge; where every care is taken to forward their views and regulate their studies, to enable them, in a short time, to pass their examinations at the College of Surgeons, and Apothecaries' Hall. Many opportunities will be allowed to those who are desirous of prosecuting their studies in the Practice of Midwifery, by attending to cases in the lower class of society. It is the wish also of the Advertiser to render this Establishment every thing that will be agreeable and pleasant in regard to domestic comfort. Further particulars may be known by application personally, or by letter, post paid, to Mr. Tucker, Surgeon, 16, Berners Street, Oxford Street.

---

I herely certify that Mr. COLES, of London Bridge, has perfectly succeeded in keeping up a very difficult and complicated rupture, on which I operated some years since, in which case many of the best truss-makers in London had failed to effect any relief.

When I placed this patient under his care, I promised, should he succeed, to give him a certificate of my approbation, and, in conformity with that, I now state my firm conviction, that his truss will be found more efficacious than any at present employed in similar cases.

I have since tried his trusses in other cases, and have been much pleased with the ingenuity he has displayed in adapting his means to the circumstances of each case. HENRY EARLE,

George Street, Hanover Square.

*July* 23, 1823.

---

Dr. POWER has in great forwardness a Second Edition of his Treatise on Midwifery; the whole being newly arranged, and comprising material alterations and additions.

---

Mr. CURTIS will commence his next Course of Lectures on the Anatomy, Physiology, and Pathology of the Ear, on the 1st of October.

Mr. R. W. Bampfield has in the press the Second Edition of his Practical Treatises on Tropical and Scorbutic Dysentery, with some additions.

### OBITUARY.

On Wednesday, 16th July, Andrew Matthias, Esq. of Old Burlington Street, Surgeon to the General Lying-in Hospital. His work on the "Mercurial Disease" obtained him deserved celebrity, and amply rewarded him for his research and close investigation. His death was occasioned by apoplexy.

On Wednesday, June 4th, John William Newby, Esq. of Poland Street, in consequence of opening the body of a child who died of enteritis and erysipelas of the abdomen. This gave rise to a pustule on the back of the left thumb, inflammation of the axilla and breast, which occasioned his death.

